# Rheumatism, Cerebral Embolism, Paralysis, Recovery

**Published:** 1874-08

**Authors:** A. F. A. King


ALCOHOLISM, RHEUMATISM, BROMO-IODISM, CEREBRAL EMBOLISM (?) APHASIA, PARALYSIS; RECOVERY.—*By A. F. A. King, M.D.*—* * * *Remarks.*—Since this case recovered, we are (and fortunately) left in doubt as to its exact pathology, and may therefore be allowed to theorize in regard to it. We presume it to have been embolism of the left middle cerebral artery. The embolic body was probably detached from the heart or aorta, and *this*, despite the absence of physical signs; for while it is true that organic changes of a certain prodigious degree can be discovered generally by physical examination, it must be admitted that lesser degrees of structural change may altogether escape detection. The embolism, we presume, was slowly disintegrated by fatty metamorphosis, before any extensive cerebral softening had taken place, though that *some* organic cerebral change had occurred was indicated by the urinary deposit.

A question of great practical importance is this: Was not the embolism really produced by the medicines employed to relieve rheumatism? In other words, did not the salivation, or the blood changes incident to it, produced by the excess of bromides and iodides, lead to the separation of an embolism that would otherwise have remained securely attached to its seat in the heart or aorta? If the truth could be known, this question, we apprehend, would have to be answered in the affirmative. In the absence of proof, we may at least enjoin additional caution while administering these two oft-used haloids for prolonged rheumatic inflammation.

